# CD47 and CD68 expression in breast cancer is associated with tumor‐infiltrating lymphocytes, blood vessel invasion, detection mode, and prognosis

**DOI:** 10.1002/cjp2.309

**Published:** 2023-01-04

**Authors:** Ying Chen, Tor Audun Klingen, Hans Aas, Elisabeth Wik, Lars A Akslen

**Affiliations:** ^1^ Centre for Cancer Biomarkers CCBIO, Department of Clinical Medicine University of Bergen Bergen Norway; ^2^ Department of Pathology Vestfold Hospital Tønsberg Norway; ^3^ Department of Pathology Oslo University Hospital Oslo Norway; ^4^ Fürst Medical Laboratory Oslo Norway; ^5^ Department of Surgery Vestfold Hospital Tønsberg Norway; ^6^ Department of Pathology Haukeland University Hospital Bergen Norway

**Keywords:** breast cancer, CD47, CD68 macrophages, lymphatic and blood vessel invasion, tumor‐infiltrating lymphocytes (TILs), mammography screening, prognosis

## Abstract

CD47 expressed on tumor cells binds to signal regulatory protein alpha on macrophages, initiating inhibition of phagocytosis. We investigated the relationships between tumor expression of CD47 and CD68 macrophage content, subsets of tumor‐infiltrating lymphocytes (TILs), and vascular invasion in breast cancer. A population‐based series of 282 cases (200 screen detected and 82 interval patients) from the Norwegian Breast Cancer Screening Program was examined. Immunohistochemical staining for CD47 and CD68 was evaluated on tissue microarray (TMA) slides. For CD47 evaluation, a staining index was used. CD68 tumor‐associated macrophages were counted and dichotomized. TIL subsets (CD45, CD3, CD4, CD8, and FOXP3) were counted and dichotomized using immunohistochemistry on TMA slides. Vascular invasion (both lymphatic and blood vessel) was determined on whole tissue slides. High CD47 tumor cell expression or high counts of CD68 macrophages were significantly associated with elevated levels of all TIL subsets (*p* < 0.02), CD163 macrophages (*p* < 0.001), blood vessel invasion (CD31 positive) (*p* < 0.01), and high tumor cell Ki67 (*p* < 0.004). High CD47 expression was associated with ER negativity (*p* < 0.001), HER2 positive status (*p* = 0.03), and interval‐detected tumors (*p* = 0.03). Combined high expression of CD47–CD68 was associated with a shorter recurrence‐free survival (RFS) by multivariate analysis (hazard ratio [HR]: 2.37, *p* = 0.018), adjusting for tumor diameter, histologic grade, lymph node status, and molecular subtype. Patients with luminal A tumors showed a shorter RFS for CD47–CD68 high cases by multivariate assessment (HR: 5.73, *p* = 0.004). This study demonstrates an association of concurrent high CD47 tumor cell expression and high CD68 macrophage counts with various TIL subsets, blood vessel invasion (CD31 positive), other aggressive tumor features, and interval‐presenting breast cancer. Our findings suggest a link between CD47, tumor immune response, and blood vessel invasion (CD31 positive). Combined high expression of CD47–CD68 was an independent prognostic factor associated with poor prognosis in all cases, as well as in the luminal A category.

## Introduction

CD47 is identified as an integrin‐associated protein on tumor cells and binds to signal regulatory protein alpha (SIRPα) on phagocytes, such as macrophages [1]. The interaction between CD47 and SIRPα is known as a ‘do not eat me’ signal and functions to inhibit phagocytosis [2, 3]. Evidence suggests that CD47 is a dominant anti‐engulfment signal on tumor cells and is overexpressed in various cancers [2, 4, 5, 6, 7, 8]. Avoiding phagocytosis by tumor‐associated macrophages (TAMs) might promote growth and metastasis of malignant tumors [9, 10]. Thus, blocking of CD47‐SIRPα binding between tumor cells and innate immune cells can increase phagocytosis of tumor cells [11].

Some reports have documented that high expression of CD47 is associated with aggressive breast cancer features and poor prognosis [12, 13, 14]. Since CD47 also binds to thrombospondin‐1 (TSP‐1), the TSP1‐CD47 interaction might influence processes other than phagocytosis, such as proliferation, apoptosis, and migration, and could also play a role in angiogenesis and inflammation [15, 16, 17]. In a previous study, we reported that tumor cell invasion into blood vessels strongly correlates with a basal‐like tumor phenotype and interval‐detected breast cancers [18]. High levels of CD163 TAMs and tumor infiltrating lymphocyte (TIL) subsets were also related to blood vessel invasion (CD31 positive) [19, 20]. Here, we examined the combined tumor expression of CD47 and levels of CD68 TAMs, with particular focus on potential relations with TIL categories, stromal elastosis, lymphatic and blood vessel invasion by tumor cells, and tumor detection mode, examined in a population‐based retrospective series from the Norwegian Breast Cancer Screening Program.

## Materials and methods

### Study population

Patients were included from Vestfold County in Eastern Norway. Vestfold comprises around 5% of the Norwegian population with approximately 230,000 inhabitants. The Norwegian Breast Cancer Screening Program involves biennial mammography in the age‐group 50–69 years and was implemented in this county in 2004. The mean age of patients at the time of diagnosis was 60 years (range 49–70 years). A total of 37,977 women participated during the study period of March 2004–June 2009, with attendance rates of 71 and 76% in the initial and second screening rounds. The definition of an interval cancer was a tumor diagnosed between two screening sessions. During this period, 204 invasive screen‐detected cancers and 85 invasive interval cancers were diagnosed. Of these, seven patients were excluded: one screening cancer had no remaining tumor tissue; one screening case was re‐classified as a malignant phyllodes tumor; and one interval case had no biopsy or surgery performed due to multiple metastases. Further, four patients (two screening and two interval cancers) had simultaneous tumors in both breasts; the tumors with the worst prognostic profile (based on the Nottingham Prognostic Index) were selected for inclusion. Overall, 200 screening cancers and 82 interval cancers were included in this study. Clinical data, tumor stage, and survival information were retrieved from patient records. Immunohistochemical (IHC) surrogate markers for molecular subtypes of breast cancer were defined and applied according to the St. Gallen consensus from 2013 [21]. The cutoff for estrogen receptor (ER) and progesterone receptor (PR) was 1% in the present study.

Information on local tumor recurrence, distant metastases, or death was recorded for the 282 patients. The median follow‐up was 138 months (range 108–168; last clinical follow‐up was August 2018). The study was approved by the Regional Ethics Committee of Eastern Norway (reference #2018/1102).

### IHC staining

Tissue microarray (TMA) slides were used in this study. Three tissue cores (diameter 1.0 mm) were extracted from paraffin‐embedded blocks with tumor tissue and inserted into TMA recipient blocks using a semi‐automated precision instrument (Minicore 3 Tissue Arrayer, Alphelys, France). In this study, a total of 226 of the 282 (80%) cases in the cohort were available for analyses on TMA sections. In 43 (15%) cases, the TMA cores had too limited tissue for evaluation, and whole sections were therefore used as substitutes. Regarding whole sections, three small areas from the tumor periphery, corresponding approximately to three TMA cores, were selected for evaluation of CD47 and CD68 expression in a similar way as used for TMA sections. In 13 additional cases (5%), tissue from the core needle biopsy (diameter 3–4 mm) was used, as in our previous studies [19, 20].

Dual IHC staining was performed on 4–5 μm standard tumor‐tissue sections. All sections were stained using the Ventana Discovery Ultra fully automated immuno‐stainer (Roche Diagnostics, Oslo, Norway) with standard protocol procedure. Pretreatment with Ventana Discovery CC1 (Roche Cat. No. 0641457001) for 64 min was performed. Anti‐CD47 antibody SP279 (Abcam ab226837; Abcam, Cambridge, UK) at dilution 1:100 was detected by anti‐Rabbit HQ (Roche Cat. No. 07017812001), followed by Discovery Anti‐HQ HRP (Roche Cat. No. 07017936001) and Discovery Purple (Roche Cat. No. 07053983001). Anti‐CD68 antibody PG‐M1 (Dako M0876, Dako, Oslo, Norway) at dilution 1:100 was detected by Discovery OmniMap anti‐Ms HRP (Roche 05269652001) followed by Discovery Teal HRP (Roche Cat. No. 08254338001). All slides were counterstained with hematoxylin. Anti‐CD47 and anti‐CD68 dual IHC staining generated purple membranous and cytoplasmic staining for CD47 and teal (turquoise) cytoplasmic staining for CD68. Primary antibodies were omitted for the negative controls. Tissues from breast cancer with known positive CD47 and CD68 expression were used as positive controls.

### Evaluation of CD47 and CD68 expression

CD47 positive cases were evaluated for staining intensity in the tumor cell membrane or cytoplasm, as well as the staining area (fraction of positive tumor cells, %). A staining index (SI, values 0–9), obtained as a product of staining intensity (0–3) and proportion of immunopositive tumor cells (<10% = 1, 10–50% = 2, and >50% = 3), was calculated for the core with the strongest expression (‘hot core’), as previously published [22]. Cases were dichotomized (based on frequency distribution analysis) as high or strong (SI 6–9; 33% of cases) versus low or weak (SI < 6; 67% of cases). CD68 positive staining was detected in the cytoplasm of TAMs and counted using an eye‐piece graticule (10 × 10 gridlines; 0.29 × 0.29 mm; total 0.084 mm^2^) in the most active target areas (‘hot spot'), as previously published [19, 20]. High expression of combined CD47(tumor)–CD68(TAM) was defined as both high tumor cell expression of CD47 (SI 6–9) and high levels (upper quartile) of CD68 TAMs observed at the tumor periphery in the stroma or within the tumor epithelium. Some variability among the three TMA cores from the same tumor was observed. Notably, the TMA core with the most ‘active areas’ and strongest expression of CD47 and CD68 (‘hot spot’ or ‘hot core’) was selected to represent each case.

For estimation of interobserver agreement, staining for CD47 and CD68 in 50 cases was evaluated by two pathologists (YC and TAK), showing good agreement with a kappa value of 0.81 for CD47 (supplementary material, Table S1). CD68 showed a somewhat lower agreement with a kappa value of 0.66 (supplementary material, Table S2).

Information on IHC evaluation of CD3, CD4, CD8, CD45, FOXP3 TILs, CD163 TAMs, D2‐40 for lymphatic vessel invasion, and CD31 for blood vessel invasion was included from previous studies for comparison with CD47 tumor expression and CD68 TAM counts [18, 19, 20]. In addition, for this study, counts derived from the CD45‐immunostained TMA sections were compared with TIL counts based on hematoxylin and eosin (H&E)‐stained whole slides (by two pathologists, YC and TAK) and following the criteria from the international TILs working group (50 random cases) [23]. This comparison showed good agreement with a kappa value of 0.75. Baseline data on ER, PR, HER2, and Ki67 for this cohort have also been previously reported [18].

### Statistics

All analyses were performed using the SPSS package, version 26.0 (IBM Corp, Armonk, NY, USA). Statistical significance (two‐sided) was considered as a *P* value less than 0.05. Continuous variables were categorized based on quartile limits. For statistical analysis, CD47 SI and CD68 TAM counts were dichotomized; high CD47 expression (SI 6–9) versus low CD47 expression (SI < 6), and high CD68 TAM counts (by upper quartile) versus low CD68 counts (others). Associations between categorical variables were assessed using Pearson's chi‐square test. The Cohen's kappa test for interobserver variability was used to examine the agreement between two observers.

A total of 282 patients were accessible for survival analyses. The end point was disease‐specific survival (DSS), i.e. the time from diagnosis to death from breast cancer, or recurrence‐free survival (RFS), i.e. the time from diagnosis to disease recurrence for patients without metastases at the time of diagnosis. Univariate survival analyses were performed using the Kaplan–Meier method (log‐rank test for differences). Prognostic values of different variables were compared by Cox proportional hazards method for multivariate analyses, using the likelihood ratio test for differences. The variables were visually examined by log‐minus‐log plots to check for proportionality before incorporation into multivariate models. Hazard ratios (HRs) and their 95% confidence interval (CI) were estimated.

## Results

### Expression of CD47 and CD68 in breast cancer

**Figure 1:**
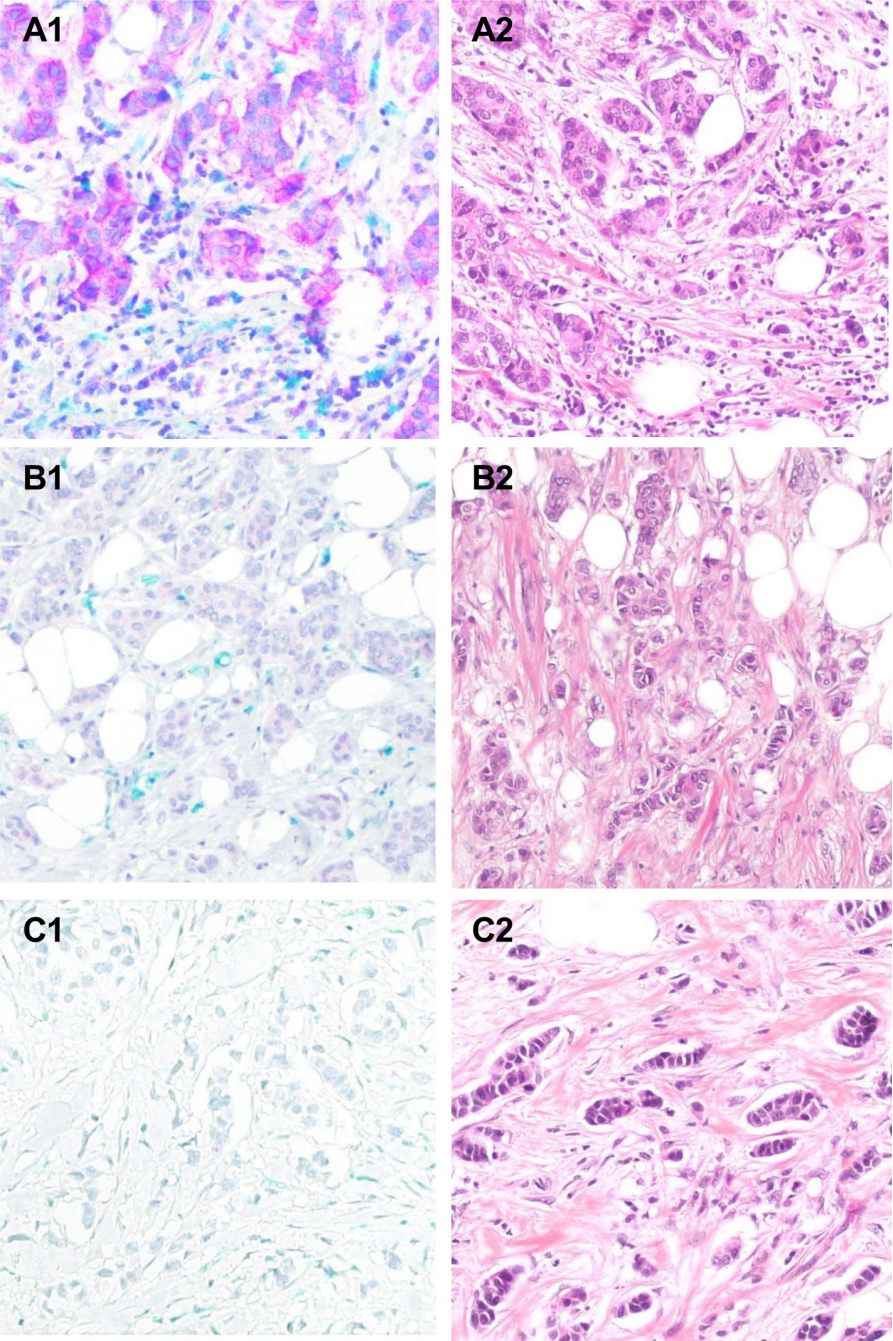
Histological images of CD47 (turquoise) and CD68 (blue) by dual IHC staining (A1, B1, C1) and H&E staining (A2, B2, C2) on the same cases of tumor tissue (×200). (A1) High expression of CD47 (score 3) and high level of CD68+ TAMs; (B1) low expression of CD47 (score 1) and low level of CD68+ TAMs; (C1) no expression of CD47 (score 0) and low level of CD68+ TAMs.

Dual IHC staining for CD47 and CD68 in tumor tissues is shown in Figure 1. Of 282 cases, 93 (33%) showed high CD47 tumor cell expression, and high counts of CD68 TAMs were found in 68 cases (24%). CD47 and CD68 showed a significant positive association (*p* < 0.001) (supplementary material, Table S3).

### Associations between CD47 or CD68, combined CD47–CD68, and clinicopathological features, vascular invasion, and breast cancer molecular subgroups

Separately, high expression of CD47, high levels of CD68‐TAMs, or combined high CD47–CD68 were significantly associated with negative ER, high Ki67, and high counts of all TIL categories (Table 1). Further, high CD47 tumor expression or high combined CD47–CD68 were associated with histologic type and interval‐detected tumors. High levels of combined CD47–CD68 were associated with low stromal elastosis.

**Table 1:** Correlation of expression of CD47, CD68, and combined CD47–CD68 with clinicopathologic features, vessel invasion, TILs, CD163 positive macrophages, elastosis, and detection mode (*n* = 282)

		CD47		CD68		CD47–CD68	
		Low	High	Low	High	Others	High
Variables	*n*	*n* (%)	*n* (%)	*n* (%)	*n* (%)	*n* (%)	*n* (%)
Histologic type							
NST	228	144 (76)	84 (90)	169 (79)	59 (87)	195 (79)	33 (97)
Others	54	45 (24)	9 (10)	45 (21)	9 (13)	53 (21)	1 (3)
		*p* = 0.005		*p* = 0.16		*p* = 0.01	
Tumor diameter							
<2 cm	214	148 (78)	66 (71)	163 (76)	51 (75)	188 (76)	26 (77)
≥2 cm	68	41 (22)	27 (29)	51 (24)	17 (25)	60 (24)	8 (24)
		*p* = 0.18		*p* = 0.84		*p* = 0.35	
Histologic grade							
1	76	57 (30)	19 (20)	65 (30)	11 (16)	73 (29)	3 (9)
2–3	206	132 (70)	74 (80)	149 (70)	57 (84)	175 (71)	31 (91)
		*p* = 0.08		*p* = 0.02		*p* = 0.01	
Lymph node status*							
Negative	187	122 (65)	65 (70)	146 (68)	41 (61)	164 (66)	23 (68)
Positive	94	66 (35)	28 (30)	68 (32)	26 (39)	83 (34)	11 (32)
		*p* = 0.40		*p* = 0.29		*p* = 0.89	
ER							
Positive	249	177 (94)	72 (77)	193 (90)	56 (82)	225 (91)	24 (71)
Negative	33	12 (6)	21 (23)	21 (10)	12 (18)	23 (9)	10 (29)
		*p* < 0.001		*p* = 0.08		*p* < 0.001	
PR							
Positive	186	129 (68)	57 (61)	144 (67)	42 (62)	167 (67)	19 (56)
Negative	96	60 (32)	36 (39)	70 (33)	26 (38)	81 (33)	15 (44)
		*p* = 0.25		*p* = 0.40		*p* = 0.19	
HER2 status							
Negative	255	176 (93)	79 (85)	197 (92)	58 (85)	227 (92)	28 (82)
Positive	27	13 (7)	14 (15)	17 (8)	10 (15)	21 (8)	6 (18)
		*p* = 0.03		*p* = 0.10		*p* = 0.09	
Ki67							
Low	211	158 (84)	53 (57)	169 (79)	42 (62)	195 (79)	16 (47)
High	71	31 (16)	40 (43)	45 (21)	26 (38)	53 (21)	18 (53)
		*p* < 0.001		*p* = 0.004		*p* < 0.001	
LVI							
Negative	212	146 (77)	66 (71)	171 (80)	41 (60)	192 (77)	20 (59)
Positive	70	43 (23)	27 (29)	43 (20)	27 (40)	56 (23)	14 (41)
		*p* = 0.25		*p* = 0.001		*p* = 0.02	
BVI							
Negative	239	169 (89)	70 (75)	188 (88)	51 (75)	217 (88)	22 (65)
Positive	43	20 (11)	23 (25)	26 (12)	17 (25)	31 (12)	12 (35)
		*p* = 0.002		*p* = 0.01		*p* = 0.002	
CD3							
Low	213	153 (81)	60 (64)	177 (83)	36 (53)	200 (81)	13 (38)
High	69	36 (19)	33 (36)	37 (17)	32 (47)	48 (19)	21 (62)
		*p* < 0.003		*p* < 0.001		*p* < 0.001	
CD4							
Low	212	148 (78)	64 (69)	168 (79)	44 (65)	193 (78)	19 (56)
High	70	41 (22)	29 (31)	46 (21)	24 (35)	55 (22)	15 (44)
		*p* < 0.08		*p* = 0.02		*p* = 0.005	
CD8							
Low	212	156 (83)	56 (60)	171 (80)	41 (63)	199 (80)	13 (38)
High	70	33 (17)	37 (40)	43 (20)	27 (37)	49 (20)	21 (62)
		*p* < 0.001		*p* < 0.001		*p* < 0.001	
CD45							
Low	212	150 (79)	62 (67)	174 (81)	38 (56)	197 (79)	15 (44)
High	70	39 (21)	31 (33)	40 (19)	30 (44)	51 (21)	19 (56)
		*p* = 0.02		*p* < 0.001		*p* < 0.001	
FOXP3							
Low	212	156 (83)	56 (60)	171 (80)	41 (60)	199 (80)	13 (38)
High	70	33 (17)	37 (40)	43 (20)	27 (40)	49 (20)	21 (62)
		*p* < 0.001		*p* < 0.001		*p* < 0.001	
CD163							
Low	212	155 (82)	57 (61)	180 (84)	32 (47)	199 (80)	13 (38)
High	70	34 (18)	36 (39)	34 (16)	36 (53)	49 (20)	21 (62)
		*p* < 0.001		*p* < 0.001		*p* < 0.001	
Elastosis							
Low	237	155 (82)	82 (88)	176 (82)	61 (90)	204 (82)	33 (97)
High	45	34 (18)	11 (12)	38 (18)	7 (10)	44 (18)	1 (3)
		*p* = 0.18		*p* = 0.14		*p* = 0.03	
Detection mode							
Screening	200	142 (75)	58 (62)	158 (74)	42 (62)	183 (74)	17 (50)
Interval	82	47 (25)	35 (38)	56 (26)	26 (38)	65 (26)	17 (50)
		*p* = 0.03		*p* = 0.06		*p* = 0.004	

*P* values were obtained using Pearson's chi‐square test. High CD3, CD4, CD8, CD45, FOXP3, CD68, CD163, and Ki67 counts are given by the upper quartile. High levels of combination of CD47–CD68 are defined both high expression of CD47 (upper tertile, SI 6–9) and high counts of CD68 (upper quartile) in the tumor tissue.

BVI, blood vessel invasion; LVI, lymphatic vessel invasion; *n*, number of cases; NST, no special type.

* One case excluded due to missing information on lymph node status.

High expression of CD47 or CD68 and combined CD47–CD68 were significantly associated with blood vessel invasion (CD31). CD68 and combined CD47–CD68 were also related to lymphatic vessel invasion (D2‐40) (Table 1). High CD47 expression and high CD68 counts were strongly associated with combined lymphatic and blood vessel invasion (Table 2).

**Table 2:** Expression of CD47, CD68, and combined CD47–CD68 by different categories of vessel invasion (*n* = 282)

	LVI−/BVI−	LVI+/BVI−	LVI−/BVI+	LVI+/BVI+	
Variables	*n* (%)	*n* (%)	*n* (%)	*n* (%)	*P* value
Total	194 (68)	45 (16)	18 (6)	25 (10)	
CD47					
High	59 (30)	11 (24)	7 (39)	16 (64)	
Low	135 (70)	34 (76)	11 (61)	9 (36)	0.004
CD68					
High	35 (18)	29 (64)	6 (33)	11 (44)	
Low	159 (82)	16 (36)	12 (67)	14 (56)	0.004
CD47–CD68					
High	17 (9)	5 (11)	3 (17)	9 (36)	
Others	177 (91)	40 (89)	15 (83)	16 (64)	0.001

Number of cases (*n*) and % within different categories of vessel invasion. *P* values were obtained using Pearson's chi‐square test. High levels of combination of CD47–CD68 are defined both high expression of CD47 (upper tertile, SI 6–9) and high counts of CD68 (upper quartile) in the tumor tissue.

BVI, blood vessel invasion; LVI, lymphatic vessel invasion.

This series showed 51% luminal A, 32% luminal B (HER2−), 6% luminal B (HER2+), 4% HER2 type, and 7% triple negative cancers. High expression of CD47, CD68, and combined CD47–CD68 was more often found in triple negative and HER2 positive categories (Table 3). Luminal A and luminal B (HER2−) tumors showed in general lower expression of CD47 or CD68 and combined CD47–CD68 (Table 3).

**Table 3:** Expression of CD47, CD68 and combined CD47–CD68 by tumor subtype (n = 282)

	Luminal A	Luminal B		HER2+	Triple negative	
		HER2−	HER2+			
Variables	*n* (%)	*n* (%)	*n* (%)	*n* (%)	*n* (%)	*P* value
Total	145 (51)	89 (32)	18 (6)	9 (4)	21 (7)	
CD47						
High	33 (23)	33 (37)	8 (44)	6 (67)	13 (62)	<0.001
Low	112 (77)	56 (63)	10 (56)	3 (33)	8 (38)	
CD68						
High	27 (19)	22 (25)	9 (50)	1 (11)	9 (43)	0.004
Low	118 (81)	67 (75)	9 (50)	8 (89)	12 (57)	
CD47–CD68						
High	8 (5)	12 (13)	5 (28)	1 (11)	8 (38)	<0.001
Others	137 (95)	77 (87)	13 (72)	8 (89)	13 (62)	

Number of cases (*n*) and % within molecular subgroups are given according to the St. Gallen consensus 2013. *P* values were obtained using Pearson's chi‐square test. High levels of combination of CD47–CD68 are defined both high expression of CD47 (upper tertile, SI 6–9) and high counts of CD68 (upper quartile) in the tumor tissue.

### Association between CD47 or CD68, combined CD47–CD68, and prognosis

Distant metastases were observed in 38 cases (13%), 6 patients had local recurrence only (2%), and 34 patients (12%) died of breast cancer during the follow‐up period.

**Figure 2:**
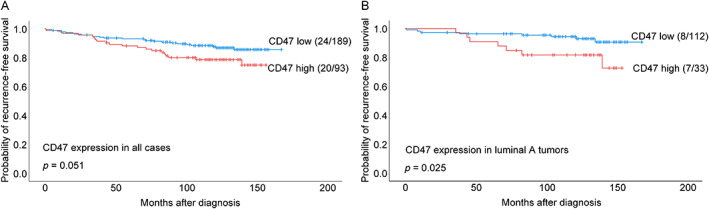
Estimated RFS according to high and low CD47 expression. Kaplan–Meier univariate RFS analysis according to CD47 protein expression (log‐rank test for difference). (A) All cases and (B) luminal A cases. For each category, the number of breast cancer recurrences is given, followed by the total number of cases in each category.

**Figure 3:**
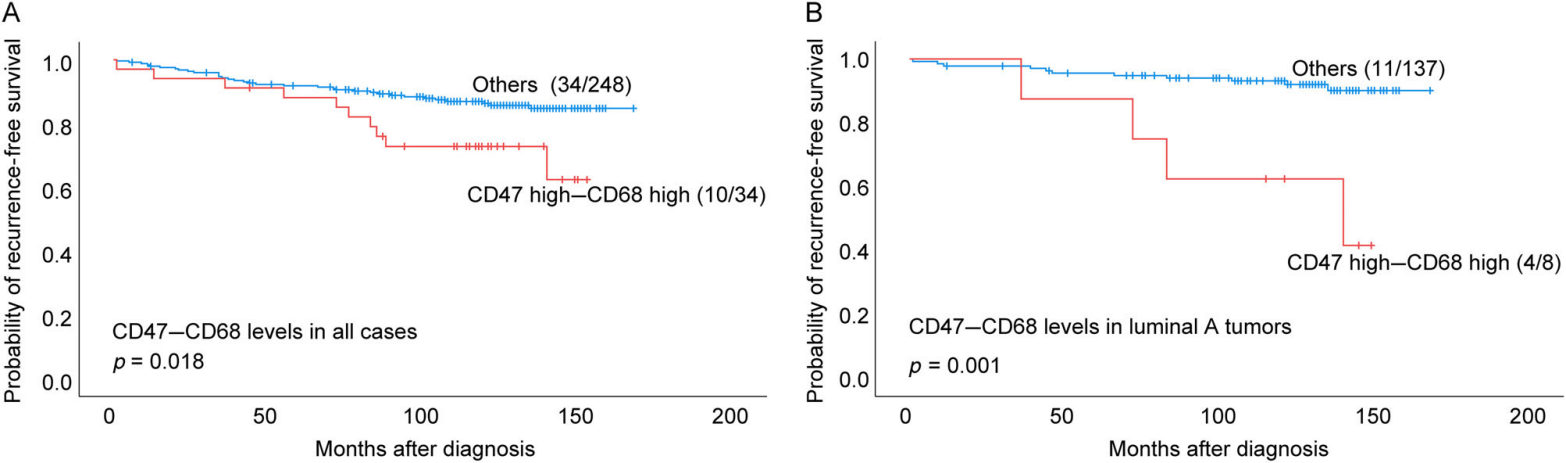
Estimated RFS according to combined CD47–CD68 levels (high versus others). Kaplan–Meier univariate survival analysis according to CD47–CD68 levels (log‐rank test for difference). RFS in (A) all cases and (B) luminal A cases. For each category, the number of breast cancer recurrences is given, followed by the total number of cases in each category.

By univariate survival analyses of all cases, high expression of CD47 alone showed a trend of association with reduced RFS (*p* = 0.051; Figure 2A). Among luminal A cases, high CD47 was associated with shorter RFS (*p* = 0.025; Figure 2B). High levels of CD68 macrophages alone were not significantly related to RFS in the whole cohort (supplementary material, Figure S1A), whereas high CD68 TAM counts were associated with shorter RFS within the luminal A subset (supplementary material, Figure S1B). We found reduced RFS for combined CD47–CD68‐high cases in all tumors (*p* = 0.018), and among luminal A cases (*p* < 0.001) (Figure 3A,B), with shorter DSS in all luminal (HER2−) tumors (*p* = 0.039). When analyzing each of the three other subgroups (of CD47–CD68 combinations) separately in comparison with the remaining cases, we found no significant differences (supplementary material, Figure S2).

**Figure 4:**
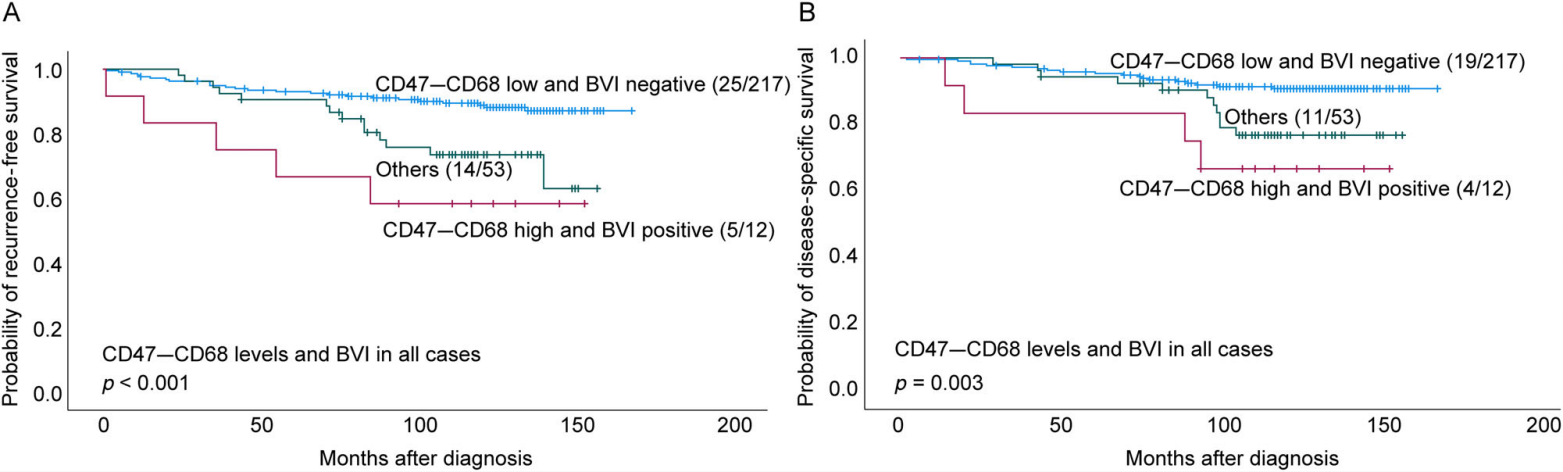
Estimated RFS and DSS by combined CD47–CD68 levels in combination with blood vessel invasion (BVI) status. Kaplan–Meier univariate survival analysis according to CD47 and BVI (log‐rank test for difference). (A) RFS and (B) DSS (all cases). For each category, the number of breast cancer recurrences or deaths from breast cancer is given, followed by the total number of cases in each category.

Cases with a combination of high CD47–CD68 and blood vessel invasion (CD31 positive) showed significantly reduced RFS (*p* < 0.001) and DSS (*p* = 0.003) compared with other cases (Figure 4A,B). Other combinations of CD47–CD68 and blood vessel invasion (CD31 positive) were not significant when compared with the rest. For combined high CD47–CD68 and lymphatic vessel invasion (D2‐40) positive cases, no significant survival difference was present.

Basic histopathological markers such as tumor diameter, histologic grade, lymph node status, and molecular subtype (including information on ER, HER2 status, and Ki67) together with CD47 or combined CD47–CD68 were included in multivariate analyses of RFS (all cases). High expression of CD47 alone, and combined high CD47–CD68, was significantly associated with shorter RFS (HR: 1.9, *p* = 0.042; HR: 2.1, *p* = 0.035) (Tables 4 and 5). High levels of CD68 macrophages alone were not significantly related to RFS by multivariate analysis (supplementary material, Table S4). Interestingly, high expression of CD47 alone and combined CD47–CD68 indicated shorter RFS among luminal A tumors by multivariate assessment (HR: 3.9, *p* = 0.012; HR: 5.7, *p* = 0.004), adjusting for tumor diameter, histologic grade, and lymph node status (Tables 6 and 7 **)**.

**Table 4:** Univariate and multivariate analysis of RFS (Cox proportional hazards method) compared with pathological variables and CD47 expression by immunohistochemistry (*n* = 282)

Variables	Categories	Univariate analysis		Multivariate analysis	
		HR (95% CI)	*P* value	HR (95% CI)	*P* value
CD47	Low	1		1	
	High	1.8 (1.0–3.3)	0.054	1.9 (1.0–3.5)	0.042
Tumor diameter	<2 cm	1		1	
	≥2 cm	2.5 (1.4–4.5)	0.003	1.1 (0.6–2.2)	0.717
Histologic grade	1	1		1	
	2–3	4.0 (1.4–11.2)	0.008	3.0 (1.0–8.7)	0.048
Lymph node status	Negative	1		1	
	Positive	3.9 (2.1–7.3)	<0.001	4.1 (2.1–8.0)	<0.001
Molecular subtypes	Luminal (HER2−)	1		1	
	HER2+/triple negative	0.6 (0.3–1.1)	0.105	0.7 (0.3–1.5)	0.359

High expression of CD47 is defined as staining index between 6 and 9 (upper tertile).

**Table 5:** Univariate and multivariate analysis of RFS (Cox proportional hazards method) compared with pathological variables and combined CD47–CD68 expression by immunohistochemistry (*n* = 282)

Variables	Categories	Univariate analysis		Multivariate analysis	
		HR (95% CI)	*P* value	HR (95% CI)	*P* value
CD47–CD68 combination	Others	1		1	
	High	2.3 (1.1–4.6)	0.021	2.4 (1.2–4.8)	0.035
Tumor diameter	<2 cm	1		1	
	≥2 cm	2.5 (1.4–4.5)	0.003	1.3 (0.7–2.4)	0.508
Histologic grade	1	1		1	
	2–3	4.0 (1.4–11.2)	0.008	3.0 (1.1–8.7)	0.064
Lymph node status	Negative	1		1	
	Positive	3.9 (2.1–7.3)	<0.001	3.8 (2.0–7.2)	<0.001
Molecular subtypes	Luminal (HER2−)	1		1	
	HER2+/triple negative	0.6 (0.3–1.1)	0.105	0.7 (0.3–1.4)	0.299

High levels of combination of CD47–CD68 are defined both high expression of CD47 (upper tertile, SI 6–9) and high counts of CD68 (upper quartile) in the tumor tissue.

**Table 6:** Univariate and multivariate analysis of RFS (Cox proportional hazards method) compared with pathological variables and CD47 expression by immunohistochemistry in luminal A cases (*n* = 145)

Variables	Categories	Univariate analysis		Multivariate analysis	
		HR (95% CI)	*P* value	HR (95% CI)	*P* value
CD47	Low	1		1	
	High	3.0 (1.1–8.3)	0.033	3.9 (1.4–11.1)	0.012
Tumor diameter	<2 cm	1		1	
	≥2 cm	5.0 (1.8–13.8)	0.002	2.4 (0.7–8.0)	0.168
Histologic grade	1	1		1	
	2–3	4.4 (1.0–19.4)	0.052	2.5 (0.5–12.5)	0.252
Lymph node status	Negative	1		1	
	Positive	4.2 (1.4–12.4)	0.011	2.7 (0.8–9.2)	0.114

High expression of CD47 is defined as staining index between 6 and 9 (upper tertile).

**Table 7:** Univariate and multivariate analysis of RFS (Cox proportional hazards method) compared with pathological variables and combined CD47–CD68 expression by immunohistochemistry in luminal A cases (*n* = 145)

Variables	Categories	Univariate analysis		Multivariate analysis	
		HR (95% CI)	*P* value	HR (95% CI)	*P* value
CD47–CD68 combination	Others	1		1	
	High	6.6 (2.1–20.8)	0.001	5.7 (1.7–19.0)	0.004
Tumor diameter	<2 cm	1		1	
	≥2 cm	5.0 (1.8–13.8)	0.002	2.3 (0.7–7.8)	0.175
Histologic grade	1	1		1	
	2–3	4.4 (1.0–19.4)	0.052	2.2 (0.5–11.0)	0.328
Lymph node status	Negative	1		1	
	Positive	4.2 (1.4–12.4)	0.011	2.3 (0.7–8.0)	0.187

High levels of combination of CD47–CD68 are defined both high expression of CD47 (upper tertile, SI 6–9) and high counts of CD68 (upper quartile) in the tumor tissue.

## Discussion

The development and progress of breast cancer is an extremely complex process, involving multiple factors in tumor cells and the supporting microenvironment, for example inflammatory and immune cells, connective tissue cells, and features such as early tumor spread by vascular invasion [24, 25]. Several prior publications suggest that overexpression of CD47 by tumor cells inhibits phagocytosis by macrophages, allowing cancer cells to evade immune surveillance, and this is associated with tumor progression in several cancer types [2, 26, 27, 28]. However, the relationship between CD47 and other microenvironment features such as TIL subtypes, and how these factors promote early tumor spread by blood vessel invasion in breast cancer tissues are less investigated.

To our knowledge, the present findings are the first to indicate that high expression of CD47 on tumor cells or combined high CD47–CD68 is significantly associated with blood vessel invasion, high levels of various TIL subsets, high CD163 TAMs, and interval tumor presentation in a population‐based mammography screening material. In line with other studies, we found that high CD47 expression and high combined CD47–CD68 are associated with features of aggressive tumors such as ER negativity, HER2 positivity, and high tumor cell proliferation by expression of Ki67. Thus, coordinated effects of CD47, TAMs, and TILs appear to enable lymphatic and blood vessel invasion and breast cancer progress.

Notably, we suggest that tumors with combined high expression of CD47 and CD68 have increased both lymphatic and blood vessel invasion. Our findings are in line with a previous study on non‐small cell lung cancer (NSCLC), investigating both human tumor tissues and cancer cell lines, indicating that high expression of CD47 and CD68 promotes tumor invasion and metastasis [5]. CD47 is known to play a role in angiogenesis, and studies demonstrate a crosstalk between CD47 and vascular endothelial growth factor receptor‐2 (VEGFR‐2) and TSP‐1, important angiogenic regulators [29, 30, 31].

CD68 macrophages polarize into two phenotypes, M1 and M2. Whereas M1 macrophages function as promotors of inflammation, M2 macrophages are immunosuppressive and promote tumor progression. CD163 M2 macrophages and a small proportion of M1 macrophages are considered as TAMs [32]. M1 macrophages can also switch to an M2‐like phenotype as the tumor begins to invade [33, 34]. Breast cancer is often infiltrated by TAMs, and an experimental model demonstrated multiple mechanisms of interaction between TAMs and tumor cells [35]. TAMs are known to produce proangiogenic factors, such as VEGF, to increase the network of vessels, and promote migration and intravasation of tumor cells into blood vessels [36, 37]. Further, TAMs release anti‐inflammatory cytokines which decrease proinflammatory T‐cell immune response and promote tumor progression and metastasis [38, 39]. These experimental observations may support our findings that CD47 and CD68 combined are strongly related to both lymphatic and blood vessel invasion in breast cancer.

Here, we found a strong association between overexpression of CD47 on tumor cells and high levels of all TIL subsets in breast cancers, supporting a link between CD47 and the innate immune system [40]. The role of TILs as prognostic markers, especially CD8 and FOXP3 subtypes, has been evaluated in large studies, the majority focusing on ER−, HER2+, and triple negative breast cancers. In these subsets, the presence of high levels of TILs has been associated with better patient survival [41, 42, 43]. However, some other studies and our previous study suggest that high TIL counts might be associated with poorer prognosis in ER+ patients [20, 44, 45, 46].

CD47 plays an important role in the immune evasion of tumor cells through direct or indirect interactions with different types of immune cells [47]. However, the underlying mechanisms are not entirely clear, and increased number of various TIL subgroups does not present a simple reflection of immune function. CD47 has a complex and multifactorial role in anti‐cancer immunity and is involved in the regulation of different immune cell activities [48]. CD47‐SIRPα participates in T‐cell recruitment at sites of inflammation *in vivo* and regulates T‐cell transendothelial migration [49]. Interaction of CD47‐SIRPα results in inhibition of phagocytosis and stimulates T‐cell activation and neutrophil transepithelial migration [16, 50]. Thus, cancer cells may escape immune cells by upregulation of CD47 expression [28].

Interestingly, our study indicates that FOXP3 has the strongest association with CD47, and infiltration of FOXP3 cells is correlated with poor prognosis in several cancers [51]. Notably, anti‐CD47 treatment may enhance anti‐tumor T‐cell immunity [52], and a recent publication on pancreatic cancer indicated that CD47 targeting induces remodeling of tumor‐infiltrating immune cells of the tumor microenvironment [53].

Several studies suggest that expression of CD47 is a prognostic marker in many different types of cancers, such as leukemia [4], lymphoma [54], hepatocellular carcinoma [55], bladder cancer [56], NSCLC [5], leiomyosarcoma [57], colorectal cancer [58], and pancreatic neuroendocrine tumors [59]. Previous reports suggest that high expression of CD47‐SIRPα in the bone marrow and peripheral blood predicts recurrence in breast cancers [60], and increased CD47 in breast stem cells inhibits phagocytosis [61]. Only a few studies report direct evidence of any correlation between the expression of CD47 and prognosis in breast cancer. A recent study showed that high CD47 expression was associated with epithelial–mesenchymal transition and poor prognosis in triple negative breast cancers [12]. Another study indicated that high levels of combined CD47–CD68 represent an independent predictor of poor prognosis in breast cancer, especially in patients with hormone receptor‐negative tumors [13]. Our findings are similar, except that we found reduced survival in the luminal A category. These results may indicate that when tumor CD47 expression is upregulated combined with increased number of macrophages, this may represent an adaptation to reduced phagocytic activity.

As mentioned, we found that high CD47–CD68 was not only an independent prognostic factor among all breast cancers, but also within the hormone receptor‐positive luminal tumors. These mainly low‐grade breast cancers are frequently detected by mammography screening [62]. It is still a challenge in clinical practice to predict who would benefit from one therapy approach over another in patients with luminal tumors, and it is therefore important to identify prognostic markers for this large subgroup, as a basis for improved patient stratification and more precise treatment. Baccelli *et al* reported that co‐expression of CD47 and MET in circulating tumor cells was associated with metastases and poor prognosis in luminal type breast cancers compared to expression of CD47 or MET alone [14]. Possibly, CD47 and MET co‐expression could provide complementary information on these luminal tumors related to escape from macrophage‐mediated phagocytosis [63]. Recent studies indicate that CD47‐SIRPα is not only a prognostic marker, but may also represent a potential target for treatment [47, 64, 65, 66, 67].

Due to tumor heterogeneity, using TMA sections to evaluate CD47 tumor expression and CD68 TAM counts by IHC is a limitation of this study. To increase representation, three cores were extracted from different locations in the periphery of invasive tumor tissue. The relatively low number of patients in subgroup analyses provided weak statistical power for survival analysis. Studies in larger breast cancer cohorts are necessary to validate the potential of CD47 or CD47–CD68 as biomarkers in breast cancer [68, 69, 70, 71]. Furthermore, although semiquantitative and subjective scoring is a way of reporting tissue biomarker levels as numerical values for robust group comparisons, this method has its limitations. Validation studies need to be performed by using quantitative image‐based methods.

## Conclusion

Our study indicates that CD47 and combined CD47–CD68 are significantly associated with high levels of several TIL subsets, blood vessel invasion (CD31 positive), and interval presentation of breast cancer in a population‐based mammography screening series. CD47 might represent an independent marker of reduced survival, including among hormone receptor‐positive luminal tumors. Our findings support that CD47 is a multifaceted actor in the tumor microenvironment and might represent a potentially relevant treatment target in breast cancer.

## Author contributions statement

YC, TAK and LAA contributed to study design, data interpretation, literature search, and writing the manuscript. YC, TAK and HA participated in data collection. YC, TAK and EW performed data analysis and generation of figures. All authors approved the final version of the manuscript.

## Supporting information


**Figure S1.** Estimated RFS according to high or low CD68 levels
**Figure S2.** Estimated RFS and DSS according to different combinations of high or low CD47 or CD68 levels
**Table S1.** Crosstabulation of interobserver analysis of CD47 expression by immunohistochemistry
**Table S2.** Crosstabulation of interobserver analysis of CD68 count by immunohistochemistry
**Table S3.** Cross‐correlations between CD47 and CD68
**Table S4.** Univariate and multivariate analysis of RFS (Cox proportional hazards method) of pathological variables and CD68 expression by IHC

## Data Availability

Individual patient data and related tumor information underlying this article cannot be shared publicly due to data privacy protection laws. However, grouped data will be shared on reasonable request to the corresponding author.
