# Supplementary material for: CD47 and CD68 expression in breast cancer is associated with tumor‐infiltrating lymphocytes, blood vessel invasion, detection mode, and prognosis
Source: J Pathol Clin Res. 2023 Jan 4;9(3):151–64. doi: 10.1002/cjp2.309 (PMC10073931; doi:10.1002/cjp2.309)
Supplement: Supplementary file 1 — Figure S1. Estimated RFS according to high or low CD68 levels Figure S2. Estimated RFS and DSS according to different combinations of high or low CD47 or CD68 levels Table S1. Crosstabulation of interobserver analysis of CD47 expression by immunohistochemistry Table S2. Crosstabulation of interobserver analysis of CD68 count by immunohistochemistry Table S3. Cross‐correlations between CD47 and CD68 Table S4. Univariate and multivariate analysis of RFS (Cox proportional hazards method) of pathological variables and CD68 expression by IHC [file CJP2-9-151-s001.pdf]

CD47 and CD68 expression in breast cancer is associated with tumor-infiltrating lymphocytes, blood vessel invasion, detection mode, and prognosis

Y Chen *et al*. *J Pathol Clin Res* https://doi.org/10.1002/cjp2.309

Supplementary Figures S1, S2
Supplementary Tables S1–S4

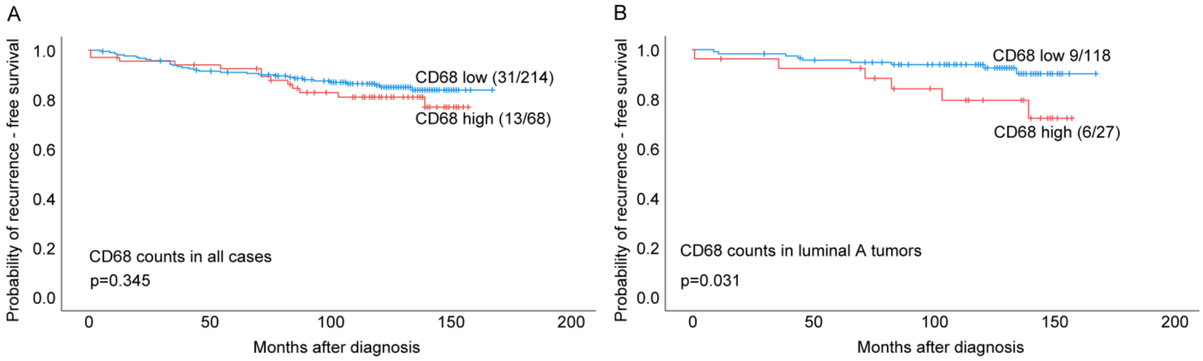

Figure S1. Estimated recurrence-free survival (RFS) according to high or low CD68 levels. Kaplan–Meier univariate survival analysis according to CD68+ TAM counts (log-rank test for difference). Recurrence-free survival in (A) all cases and (B) luminal A cases. For each category, the number of breast cancer recurrences is given, followed by the total number of cases in each category.

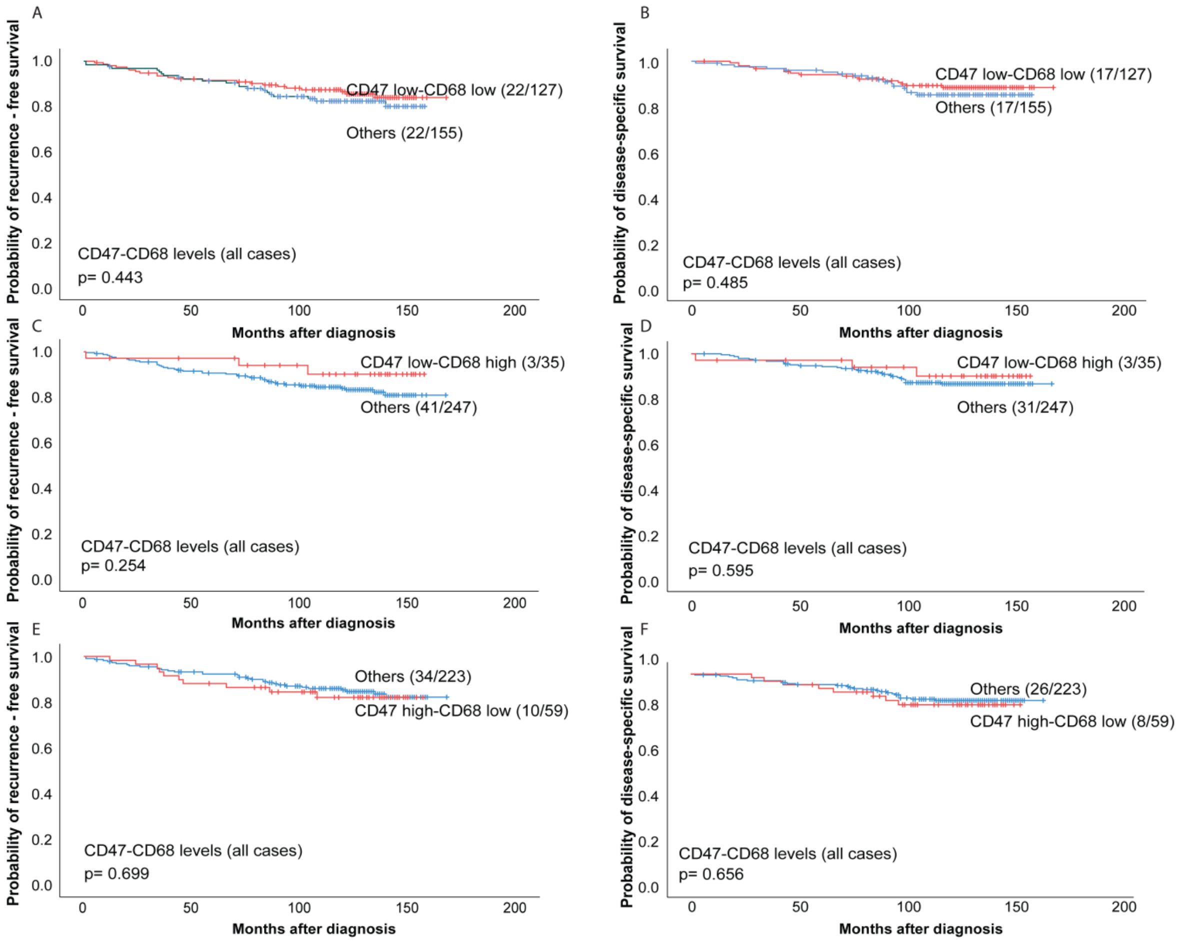

Figure S2. **Estimated recurrence-free survival (RFS) and disease-specific survival (DSS) according to different combinations of high or low CD47 or CD68 levels.**
Kaplan–Meier univariate survival analysis according to CD47 and CD68 levels (log-rank test for difference). CD47 low / CD68 low versus others: (A) RFS and (B) DSS. CD47 low/CD68 high versus others: (C) RFS and (D) DSS. CD47 high/CD68 low versus others: (E) RFS and (F) DSS. For each category, the number of breast cancer recurrences is given, followed by the total number of cases in each category.

Table S1. Crosstabulation of inter-observer analysis of CD47 expression by immunohistochemistry (*n* = 50), kappa value 0.81, *p* < 0.001

CD47 expression		Observer B					
		**0**	**1**	**3**	**6**	**9**	**total**
**Observer A**	**0**	20	1	0	0	0	21
	**1**	3	1	0	0	0	4
	**3**	0	0	8	0	0	8
	**6**	0	0	0	12	3	15
	**9**	0	0	0	0	2	2
**Total**		23	8	8	12	5	50

Kappa statistics were used in analyses regarding inter-observer agreement. CD47 positive cases were evaluated using a staining index, SI (values 0–9), obtained as a product of staining intensity (0–3) and proportion of immunopositive tumor cells (<10% = 1, 10–50% = 2, and >50% = 3) in the tumor tissue.

Table S2. Crosstabulation of interobserver analysis of CD68 count by immunohistochemistry (*n* = 50), kappa value 0.66, *p* < 0.001

CD68 count		Observer B		
		**0**	**1**	**total**
**Observer A**	**0**	32	3	35
	**1**	4	11	15
**Total**		36	14	50

Kappa statistics were used in analyses regarding inter-observer agreement. CD68 staining positive cells were counted using eye-piece graticule in the most active target areas in the tumor tissue. High levels of CD68 are defined as upper quartile (1) versus others (0).

Table S3. Cross-correlations between CD47 and CD68 (*n* = 282), *p* < 0.001

		CD68		
		**0**	**1**	**total**
**CD 47**	**0**	155	34	189
	**1**	59	34	93
**Total**		214	68	282

High levels of CD47 (1) are defined as high expression of CD47 (upper tertile, SI 6–9) versus others (0), and high levels of CD68 (1) are defined as upper quartile versus others (0). *P* values were obtained using Pearson's chi-square test.

Table S4. Univariate and multivariate analysis of recurrence-free survival (Cox proportional hazards method) versus pathological variables and CD68 expression by immunohistochemistry (*n* = 282)

Variables	Categories	Univariate analysis		Multivariate analysis	
		**HR (95% CI)**	***P* value**	**HR (95% CI)**	***P* value**
**CD68**	**Low**	1		1	
	**High**	1.4 (0.7–2.6)	0.348	1.0 (0.5–2.0)	0.978
**Tumor diameter**	**<2 cm**	1		1	
	**≥2 cm**	2.5 (1.4–4.5)	0.003	1.3 (0.7–2.4)	0.465
**Histologic grade**	**1**	1		1	
	**2–3**	4.0 (1.4–11.2)	0.008	2.9 (1.0-8.5)	0.052
**Lymph node status**	**Negative**	1		1	
	**Positive**	4.0 (2.1–7.3)	<0.001	3.7 (1.9–7.1)	<0.001
**Molecular subtypes**	**Luminal (HER2−)**	1		1	
	**HER2+/triple negative**	0.6 (0.3–1.1)	0.105	0.6 (0.3–1.2)	0.157

HR, hazard ratio; 95% CI; 95% confidence interval. High levels of combination of CD47-CD68 are defined both high expression of CD47 (upper tertile, SI 6–9) and high counts of CD68 (upper quartile) in the tumor tissue.
